# Supplementary material for: Rapid intra-host diversification and evolution of SARS-CoV-2 in advanced HIV infection
Source: Nat Commun. 2024 Aug 22;15:7240. doi: 10.1038/s41467-024-51539-8 (PMC11341811; doi:10.1038/s41467-024-51539-8)
Supplement: Supplementary file 1 — Supplementary Information [file 41467_2024_51539_MOESM1_ESM.pdf]

Supplementary Information

Rapid Intra-host Diversification and Evolution of SARS-CoV-2 in Advanced HIV Infection

Sung Hee Ko[1*], Pierce Radecki[1*], Frida Belinky[1], Jinal N. Bhiman[2,3], Susan Meiring[2], Jackie Kleynhans[2,4], Daniel Amoako[2,5], Vanessa Guerra Canedo[1], Margaret Lucas[1], Dikeledi Kekana[2], Neil Martinson[6,7], Limakatso Lebina[6], Josie Everatt[2], Stefano Tempia[2,4], Tatsiana Bylund[1], Reda Rawi[1], Peter D. Kwong[1], Nicole Wolter[2,8], Anne von Gottberg[2,8], Cheryl Cohen[2,4], Eli A. Boritz[1]

[1]Vaccine Research Center, National Institute of Allergy and Infectious Diseases, National Institutes of Health, Bethesda, MD 20892, USA

[2]National Institute for Communicable Diseases, a division of the National Health Laboratory Service, Johannesburg, South Africa

[3]SAMRC Antibody Immunity Research Unit, Faculty of Health Sciences, University of the Witwatersrand, Johannesburg, South Africa

[4]School of Public Health, Faculty of Health Sciences, University of the Witwatersrand, Johannesburg, South Africa

[5]Department of Integrative Biology and Bioinformatics, College of Biological Sciences, University of Guelph, Ontario, Canada

[6]Perinatal HIV Research Unit, University of the Witwatersrand, Johannesburg, South Africa

[7]Johns Hopkins University, Center for TB Research, Baltimore, MD 21218, USA

[8]School of Pathology, Faculty of Health Sciences, University of the Witwatersrand, Johannesburg, South Africa

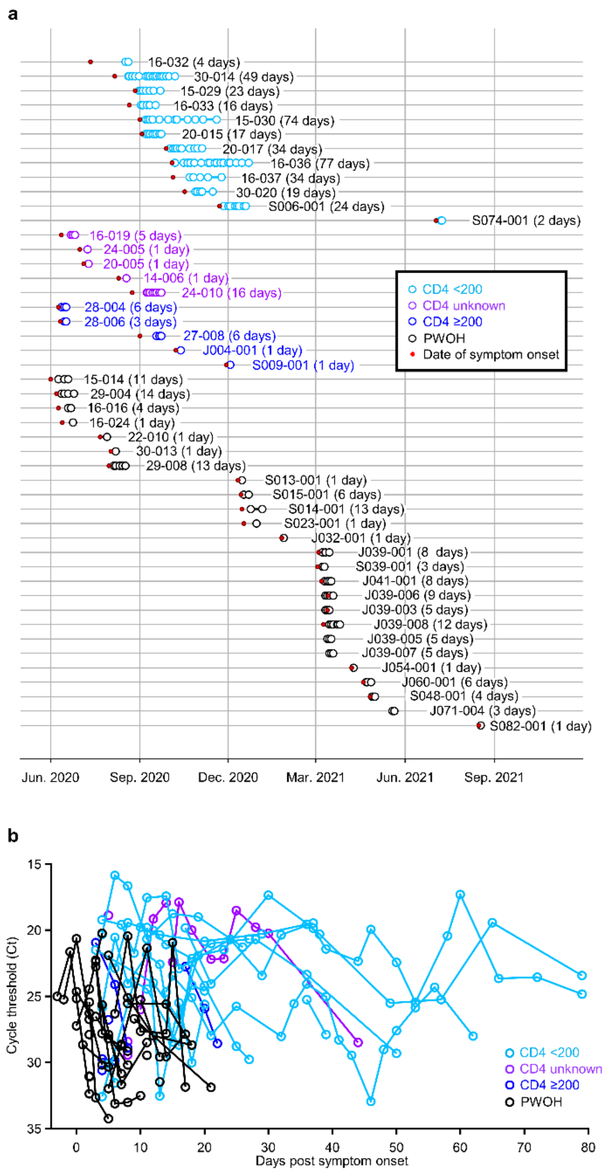

Supplementary Fig. 1. Respiratory sampling in PWH and PWOH. (a) Sampling timelines for all participants. Next to each participant identifier, the time between the first and last sequenced sample (i.e., the sequenced sampling duration) is indicated. (b) SARS-CoV-2 RNA levels (rRT-PCR Ct values) over time. Individual samples from longitudinal sample sets in each person are represented by separate datapoints. Source data are provided as a Source Data file.

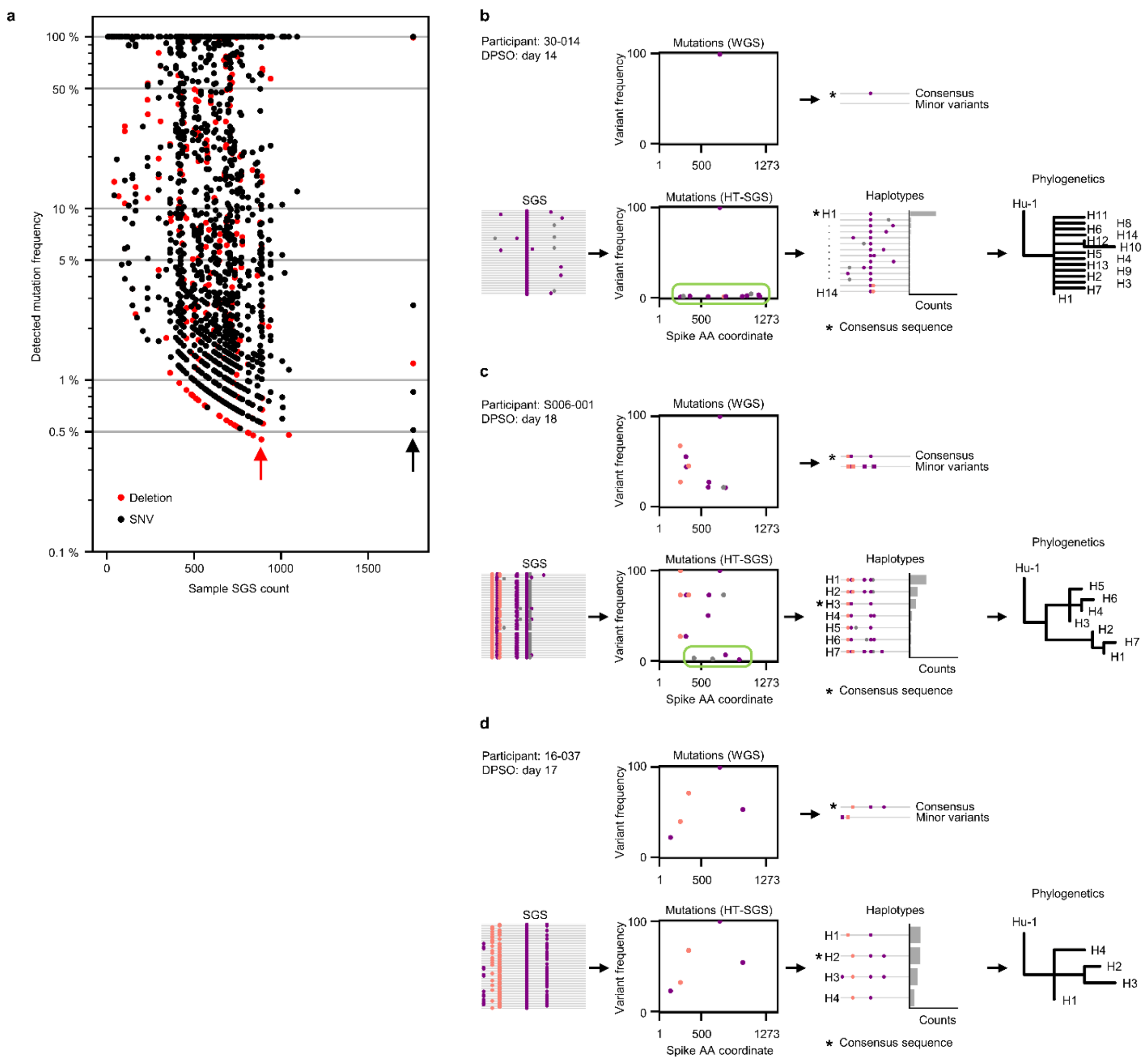

Supplementary Fig. 2. HT-SGS vs. standard whole-genome sequencing (WGS). (a) Frequencies of intra-host mutations detected by HT-SGS as a function single-genome sequence (SGS) numbers for all samples sequenced. The lowest-frequency mutation of each type detected among all samples in the study is indicated by an arrow. (b-d) Comparisons of results obtained from standard, short-read-based WGS (upper half of each panel) and HT-SGS (lower half of each panel) for three selected samples. Participant identifiers and sample timepoints (in days post symptom onset [DPSO]) are indicated. For WGS, consensus sequences and minor variant mutations are indicated. For HT-SGS, all detected haplotypes and as well as phylogenies that relate haplotypes to one another are shown. Source data are provided as a Source Data file.

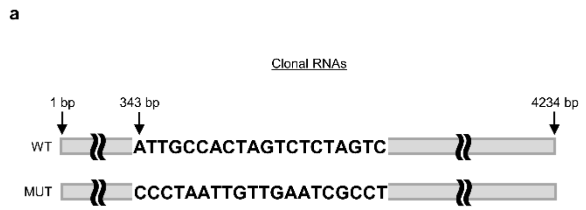

b

Template Sequence	SGS Recovered	SGS Classified as Template	Called Minor Variants
WT	2503	2503	0
Mutant	751	751	0

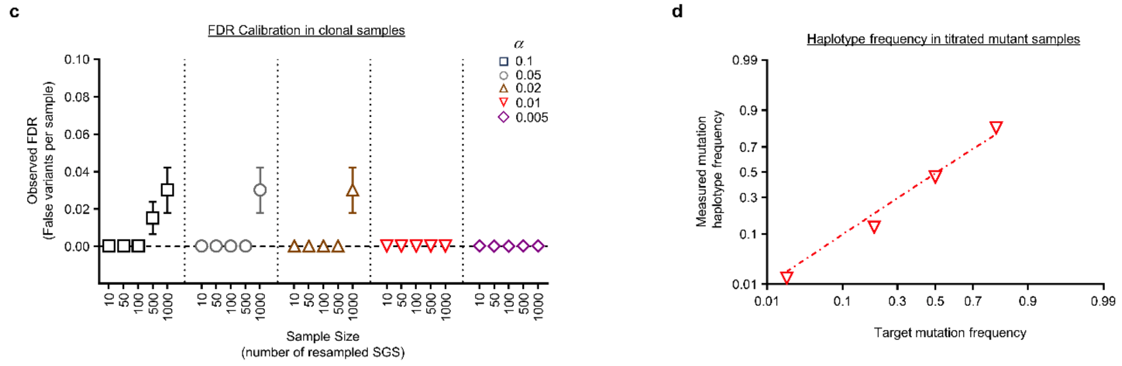

Supplementary Fig. 3. Verification of HT-SGS variant calling thresholds. (a) Diagram of wild-type (WT) and mutated (MUT) SARS-CoV-2 spike constructs used for verification of variant calling. Coordinates are provided relative to the start of the 4234-bp amplicon target used in this study. (b) Numbers of SGS recovered from each construct after in vitro transcription of RNA, HT-SGS, and mutation/haplotype calling. (c) Observed false discovery rates (FDRs) on resampled datasets from the SGS acquired in (b) across a range of minimum significance thresholds (α). For each sample size, 200 resamplings were performed (100 replicates from WT SGS and 100 replicates from MUT SGS). FDR at each sample size was calculated as the total number of minor variants called over all replicates divided by 200. The significance threshold used in our study was 0.01 (see "Variant and haplotype calling" in the Methods of main text), which yielded zero called variants across all resampled datasets (implying an FDR less than 1 variant per 1000 samples). (d) Relationship between target mutation frequency (fraction of MUT vs. WT RNA) and measured mutation frequency after HT-SGS and haplotype calling. Source data are provided as a Source Data file.

Note to Supplementary Fig. 3

Verification of HT-SGS variant calling

In addition to comparisons with corresponding short-read data, we validated the precision of our methodology with control experiments using in vitro transcribed RNA derived from plasmid constructs. First, we sequenced RNA containing either WT or MUT forms of spike. HT-SGS recovered 2503 and 751 SGS from RNA material obtained from WT and MUT plasmids (Supplementary Fig. 3a-b). Variant calling on these data at the significance threshold (α) used in our study resulted in zero called mutations and, subsequently, no minor haplotypes. We performed resampling of these data to estimate a variant false-discovery rate (FDR) for our approach. After analyses of 1000 resampled datasets, our pipeline called zero variants, demonstrating the stringency of the approach via an estimated FDR less than 1 false variant per 1000 samples processed (Supplementary Fig. 3c). Conversely, on positive controls containing titrated ratios of WT to MUT RNA, our method accurately quantified a minor variant present at a frequency of 0.2% (Supplementary Fig. 3d). These results demonstrate the sensitivity and precision of our sequencing approach for calling minor variant mutations.

a

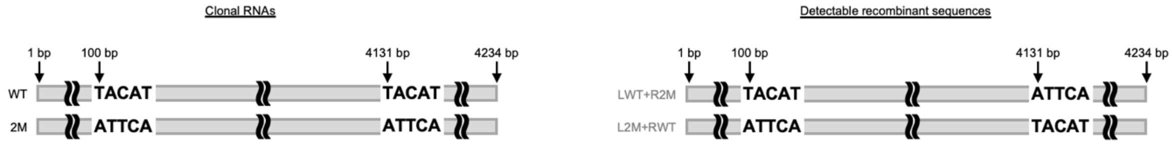

b

UMI	Mixing Step	cDNA Loaded	SGS Recovered	WT SGS	2M SGS	Recombinant SGS	Called Recombinant Haplotypes
8N	Before RT	500	59	15	43	1	0
8N	Before PCR	500	12	6	6	0	0
8N	Before RT	1500	251	52	199	0	0
8N	Before PCR	1500	196	121	75	0	0
Known	Before RT	500	10	2	8	0	0
Known	Before PCR	500	60	3	57	0	0
Known	Before RT	1500	111	83	28	0	0
Known	Before PCR	1500	132	23	109	0	0
Total			831	305	525	1	0

c

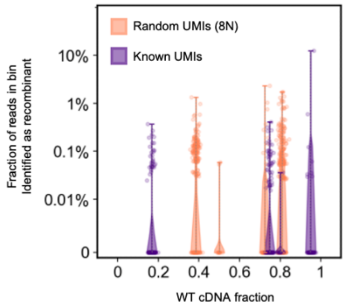

Supplementary Fig. 4. Assessment of technical recombination artifacts in HT-SGS data. (a) Diagram of wild-type (WT) and double-mutated (2M) constructs used for verification of recombination error modes in HT-SGS. Left-wild-type (LWT) + right-double-mutant (R2M) and left-double-mutant (L2M) + right-wild-type (RWT) correspond to putative technical recombinant sequences from mixed populations of WT and 2M RNA. (b) Results of HT-SGS performed on mixtures of WT and 2M RNA ("before RT" rows) and cDNA ("before PCR" rows). 8N = random 8-base UMIs; Known = whitelisted UMI pool. Replicates using whitelisted UMIs were performed to verify analyses when computing bin-level statistics of raw sequencing reads. 1 recombinant SGS was detected across the 8 samples. (c) Violin plots showing fractions of reads within bins determined to represent PCR-mediated recombinants for the 8 samples sequenced in (b). The violins indicate median values at zero in all samples, and the extent of each violin corresponds to the range of recombinant fractions observed in that sample. Source data are provided as a Source Data file.

Note to Supplementary Fig. 4

Assessment of technical recombination artifacts in HT-SGS data

To investigate potential effects of technical recombination on our findings in this study, we performed control experiments using in vitro transcribed RNA derived from WT and 2M spike plasmid constructs with anchor mutations near the 5' and 3' ends of the gene (Supplementary Fig. 4a). The positions of the mutations in the 2M construct enable the detection of sequences that are recombinants of WT and 2M spike. HT-SGS on mixtures of WT and 2M spike RNAs recovered 831 SGS over 8 samples, of which 1 SGS was deemed recombinant, corresponding to an estimated recombination rate of 0.24% at the SGS level (Supplementary Fig. 4b). Analysis of raw reads in SGS bins revealed that recombinant sequences rarely make up more than 1% of reads in a bin, demonstrating that PCR-mediated recombination is not a significant cause of errors after binning and single-copy consensus formation at the SGS level (Supplementary Fig. 4c). Varying the number of cDNAs loaded into PCR did not significantly affect the abundance of recombinant reads, nor did mixing sample material before reverse transcription ("before RT") or after ("before PCR") (Supplementary Fig. 4b-c). Replicate experiments using a pool of 354 whitelisted UMIs in place of the normal 8N (random) UMI approach gave similar results (Supplementary Fig. 4c). Overall, these findings demonstrate that technical recombination artifacts can arise during RT and PCR steps of HT-SGS processing, but at low levels. Moreover, our stringent filters for calling mutations and haplotypes ensure the fidelity of data used in downstream analyses.

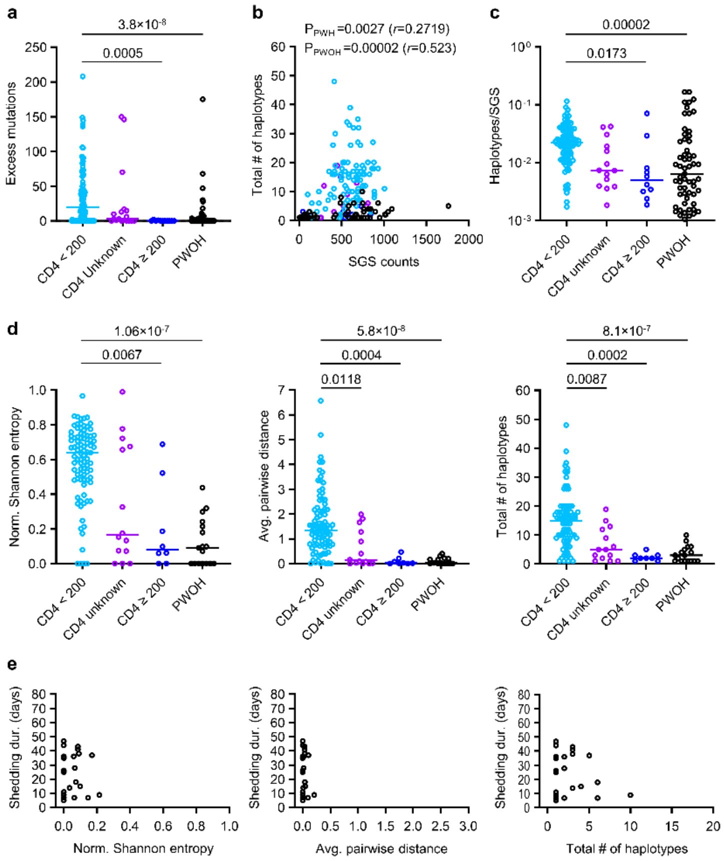

Supplementary Fig. 5. Correlations between intra-host spike diversity and multiple variables in PWH and PWOH. (a) Excess low-frequency mutations below the detection limit for each sample sequenced in the study. Excess mutations are defined as the estimated number of uncalled real mutations remaining in a sample after considering the number of called real variants and an estimated number of technical errors, calculated as described in Methods (sample size: n =66 in CD4 <200, n =11 in CD4 ≥200, n =18 in CD4 unknown , n =98 in PWOH). Statistical significance was assessed by one-way ANOVA with multiple comparisons (Kruskal-Wallis test and Dunn's multiple comparisons test); p values <0.05 are shown. (b) Numbers of haplotypes detected across individual samples as a function of SGS counts. Two-tailed Spearman p values for correlations between haplotype numbers and SGS counts are shown for PWH and PWOH; p values <0.05 are shown. (c) Ratios of haplotypes identified per SGS. Individual samples from longitudinal sample sets in each person are represented by separate datapoints (sample size: n =96 in CD4 <200, n =10 in CD4 ≥200, n =14 in CD4 unknown , n =59 in PWOH). Statistical significance was assessed by one-way ANOVA with multiple comparisons (Kruskal-Wallis test and Dunn's multiple comparisons test); p values <0.05 are shown. (d) Comparison of spike genetic diversity among

PWH subgroups and PWOH in the hospitalized cohort. Individual samples from longitudinal sample sets in each person are represented by separate datapoints (sample size: n =88 in CD4 <200, n =8 in CD4 ≥200, n =14 in CD4 unknown , n =17 in PWOH). Statistical significance was assessed by one-way ANOVA with multiple comparisons (Kruskal-Wallis test and Dunn's multiple comparisons test); p values <0.05 are shown. (e) Correlations between measurements of spike diversity at the first sample timepoint and SARS-CoV-2 RNA shedding duration in PWOH. Each datapoint represents an individual sample of each person. Statistical significance was assessed by two-tailed Spearman's rank correlation test. For all three correlations shown, p values were >0.05. Source data are provided as a Source Data file.

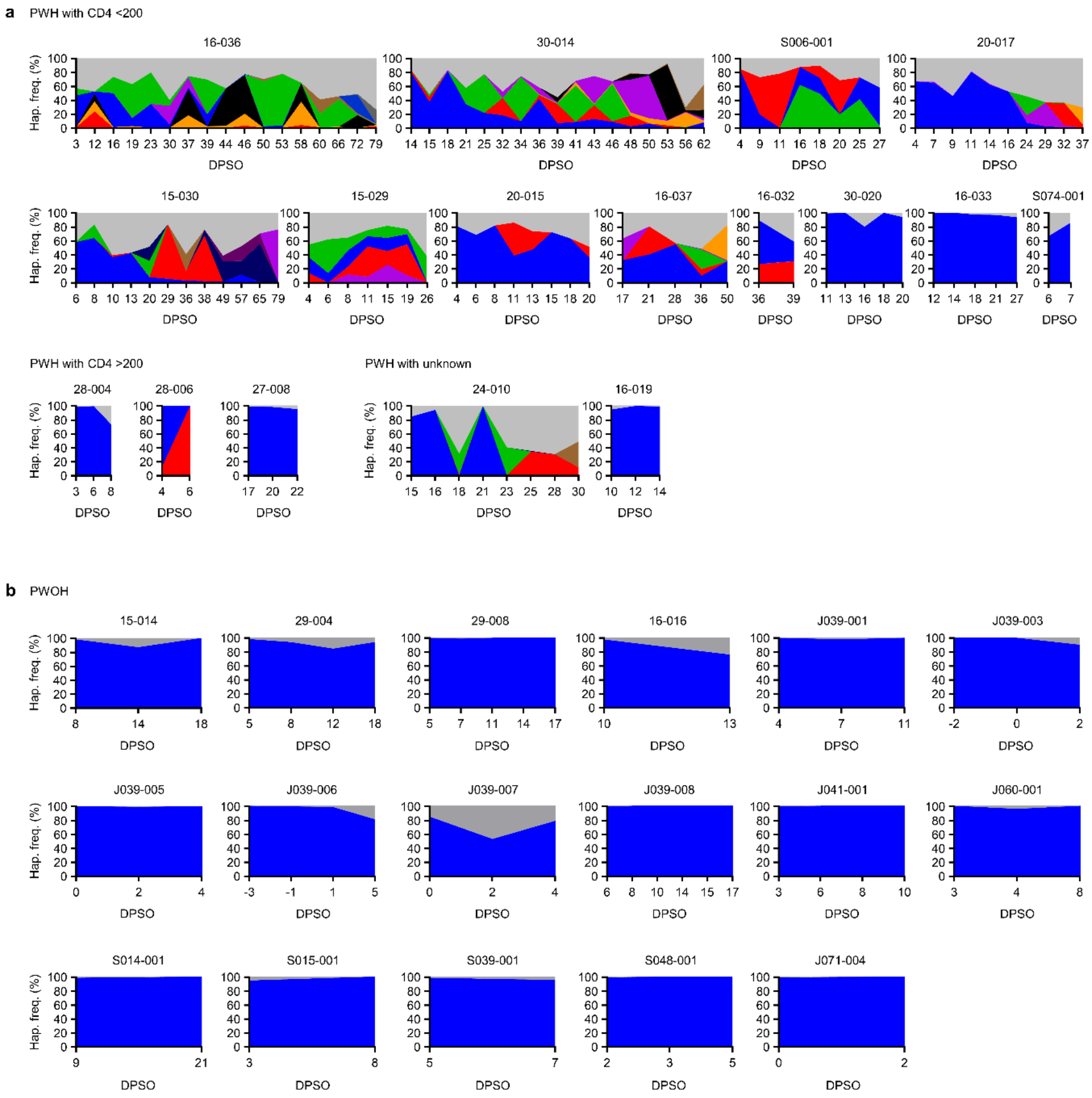

Supplementary Fig. 6. Frequencies of abundant spike haplotypes in each PWH or PWOH over time. The frequency of the haplotype that was most abundant at the first timepoint in each participant is indicated in blue, with time indicated on the x-axis as DPSO. Frequencies of other haplotypes that were most abundant in subsequent timepoints are shown with distinct colors. Grey shaded areas represent summed frequencies of all other detected haplotypes. Results are shown for PWH (a) and PWOH (b). Participants with HT-SGS data limited to one sample timepoint are excluded. Source data are provided as a Source Data file.

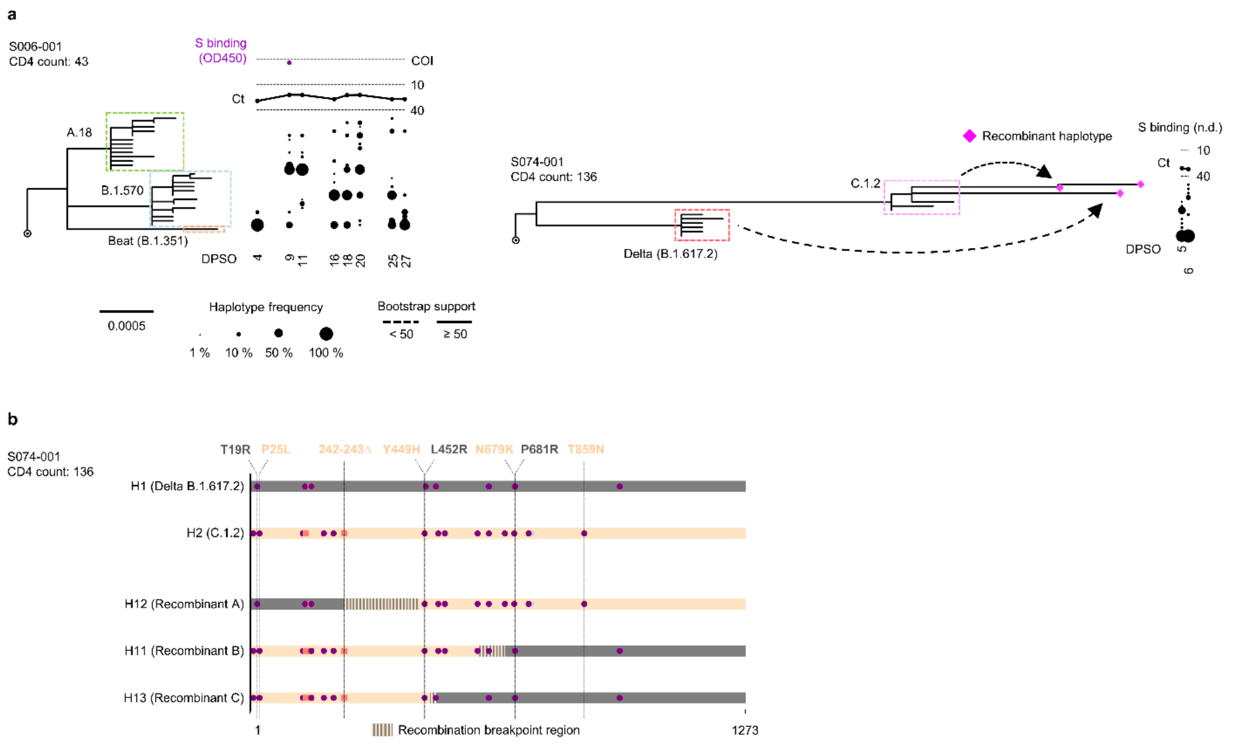

Supplementary Fig. 7. Multiple founder sequences and intra-host recombination in some PWH with CD4 counts <200 cells/μL. (a) Maximum-likelihood phylogenetic trees rooted on Hu-1 for all haplotypes from the two PWH with CD4 counts <200 cells/μL who were infected with multiple founders, as determined via single-linkage phylogenetic clustering with TreeCluster[1]. Sequences from participant S074-001 that were detected as intra-host recombinants using 3SEQ[2] are indicated. The frequency of each haplotype detected at each sample timepoint in each participant is indicated to the right of the tree via dot plot. SARS-CoV-2 RNA levels (rRT-PCR Ct values) and serum antibody binding to spike protein (optical density, 450 nm [OD450]; n.d.-no data) are shown above the dot plot for each participant. The positivity cutoff index (COI) of 0.4 for serum antibody binding to spike protein is indicated. (b) Bar-plot of three haplotypes in participant S074-001 that were deemed as intra-host recombinants. Mutations relative to Wuhan-Hu-1 are indicated. Bar segments with vertical stripes represent the inferred breakpoint regions for each recombinant haplotype.

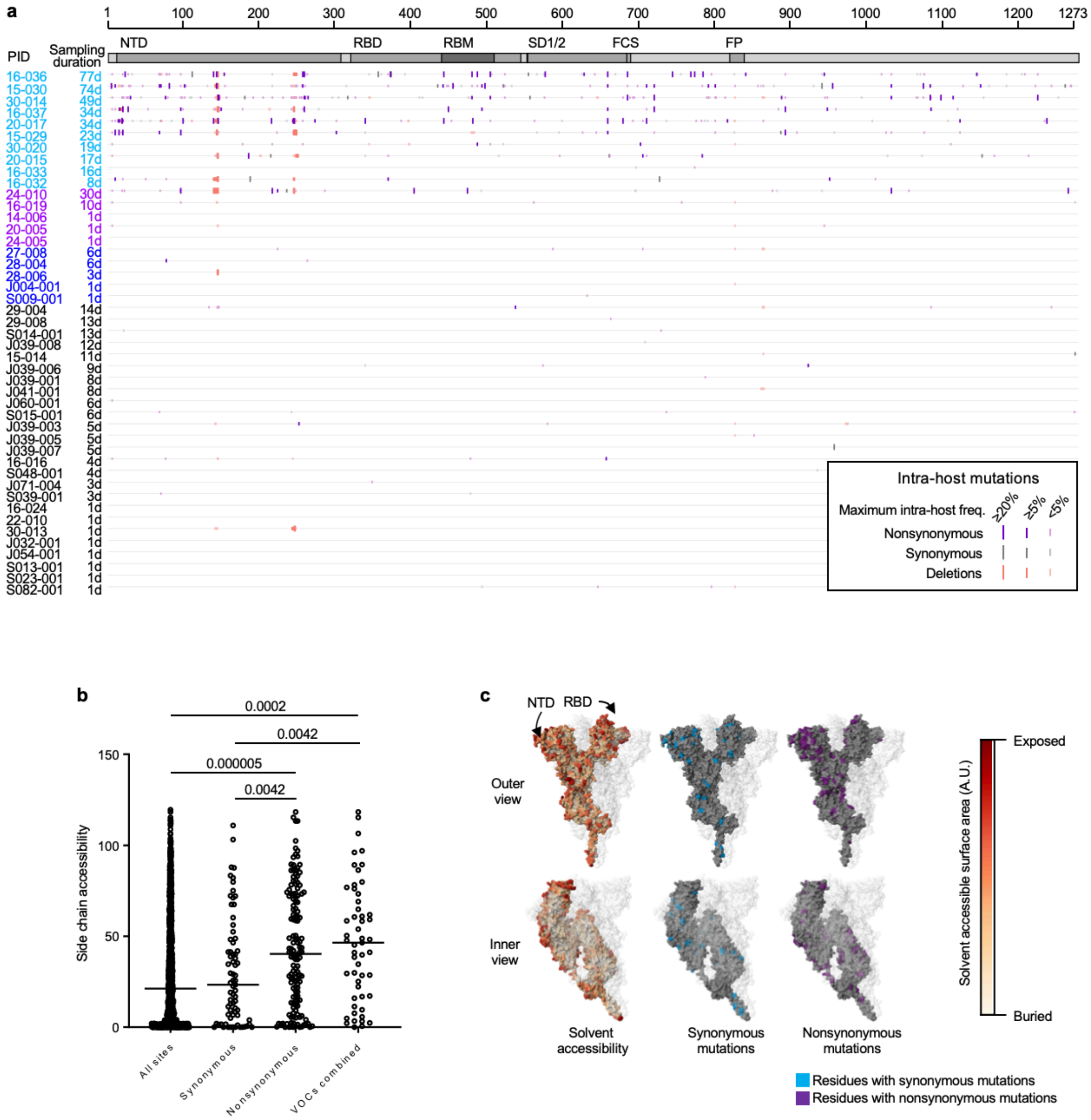

Supplementary Fig. 8. Linear and structural analysis of intra-host spike mutations in PWH and PWOH. (a) Locations and types of intra-host spike mutations detected over all timepoints in PWH and PWOH. The maximum measured frequency of each mutation is indicated by the size and color of the bar. (b) Measured side chain accessibility (i.e., side chain accessibility, A.U.) of amino acid residues in a structure model of the spike trimer. The "All sites" column refers to all 1273 amino acid residues in the spike protomer. "Synonymous" and "Nonsynonymous" columns refer to residues with intra-host synonymous (n = 73) and nonsynonymous (n = 142) mutations, respectively, in PWH with CD4 counts <200 cells/µL. The "VOCs combined" column refers to residues that are mutated in Alpha, Beta, Delta, and/or Omicron BA.1 VOCs (n = 53). Statistical

significance was assessed by one-way ANOVA with multiple comparisons (Kruskal-Wallis test and Dunn's multiple comparisons test); p values <0.05 are shown. (c) Structural representation of side chain accessibility (left), synonymous mutations (middle), and nonsynonymous mutations (right) observed in PWH with CD4 counts <200 cells/μL. Results are shown on one spike protomer, with the other two protomers of the trimer made transparent. Source data are provided as a Source Data file.

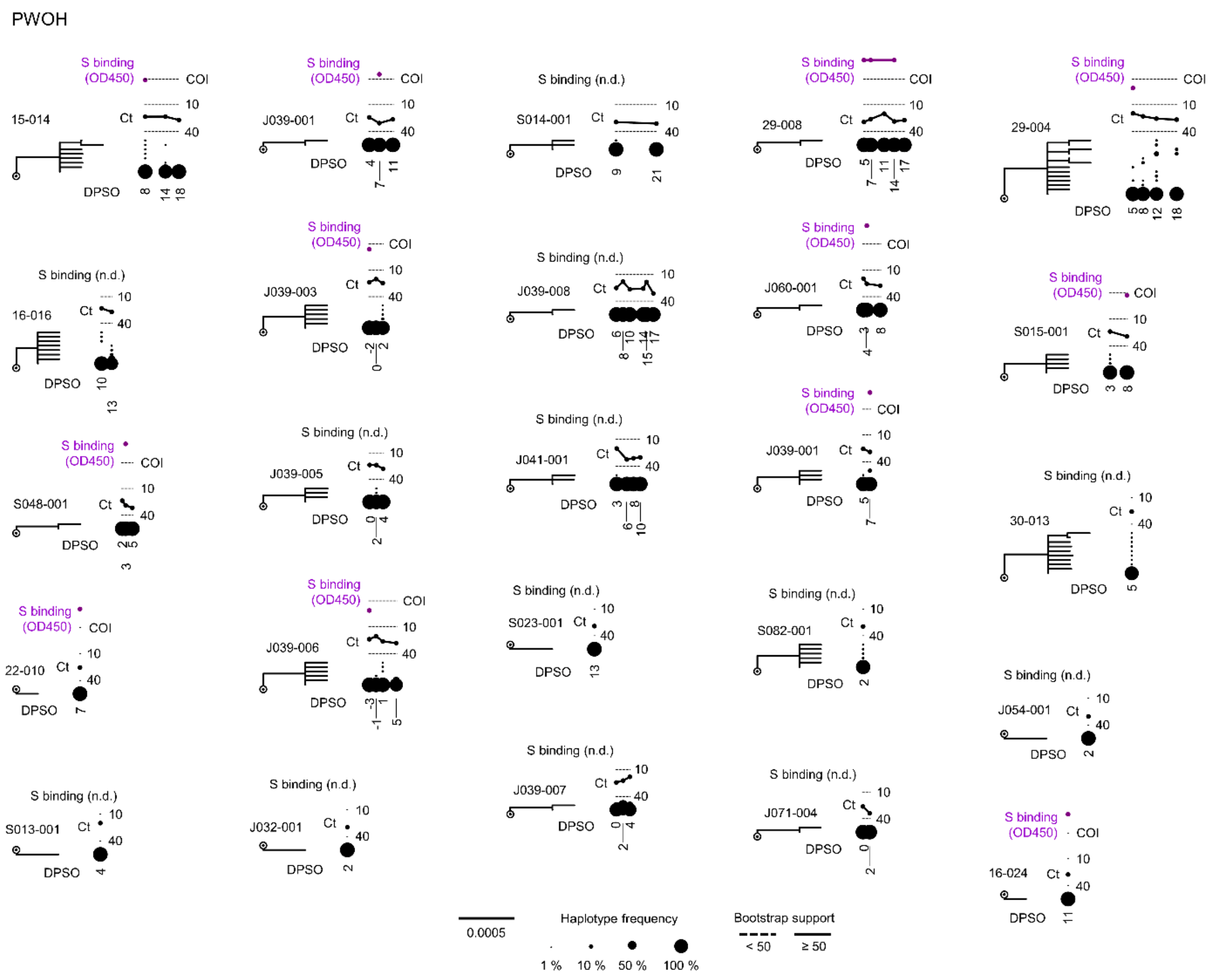

Supplementary Fig. 9. SARS-CoV-2 spike evolution in PWOH. Maximum-likelihood phylogenetic trees rooted on Hu-1 for all spike haplotypes from each PWOH. The frequency of each haplotype detected at each sample timepoint (in DPSO) in each participant is indicated to the right of the tree via dot plot. SARS-CoV-2 RNA levels (rRT-PCR Ct values) and serum antibody binding to spike protein (optical density, 450 nm [OD450]; n.d.-no data) are shown above the dot plot for each participant. The positivity cutoff index (COI) of 0.4 for serum antibody binding to spike protein is indicated.

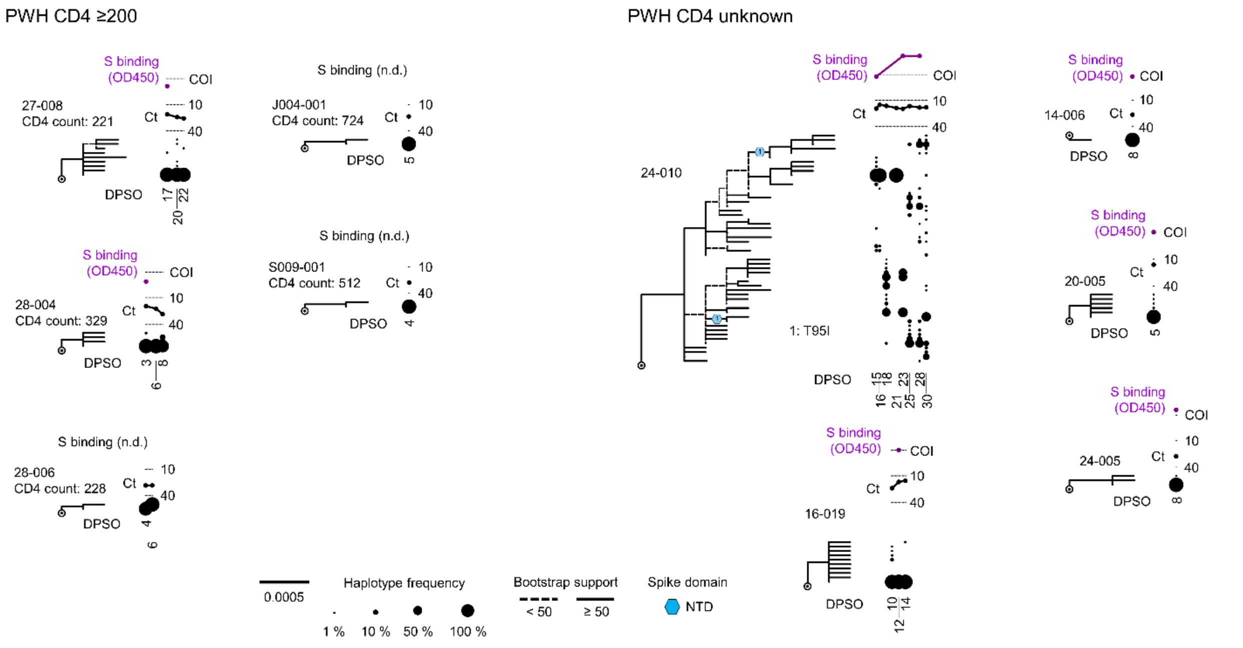

Supplementary Fig. 10. SARS-CoV-2 spike evolution in PWH with CD4 counts ≥200 cells/µL or unknown CD4 counts. Maximum-likelihood phylogenetic trees rooted on Hu-1 for all haplotypes from each PWH with CD4 count ≥200 cells/µL (left) or unknown CD4 count (right).. Sites detected under positive selection within each participant (see Methods) are shown at their inferred location on the tree with numbered symbols; mutations corresponding to each number are listed beside each participant's tree. Symbol shapes are coded by spike protein domain (see legend, center bottom). The frequency of each haplotype detected at each sample timepoint (in DPSO) in each participant is indicated to the right of the tree via dot plot. SARS-CoV-2 RNA levels (rRT-PCR Ct values) and serum antibody binding to spike protein (optical density, 450 nm [OD450]; n.d.-no data) are shown above the dot plot for each participant. The positivity cutoff index (COI) of 0.4 for serum antibody binding to spike protein is indicated.

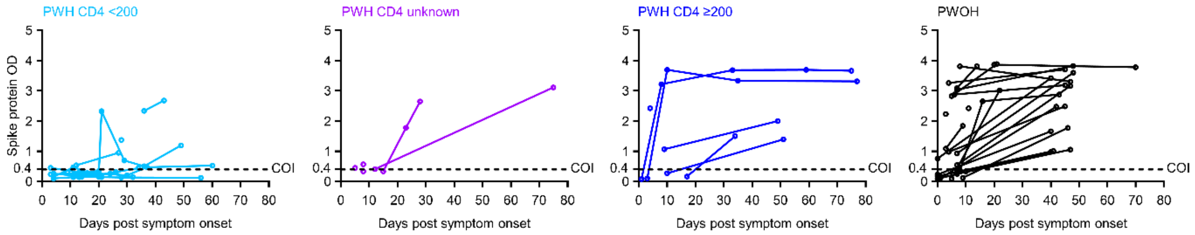

Supplementary Fig. 11. SARS-CoV-2 spike serum antibody binding responses in PWH and PWOH. Spike antibody binding titer over time in PWH subgroups and PWOH. The positivity cutoff index (COI) of 0.4 for serum antibody binding to spike protein is indicated with a dashed line. Individual samples from longitudinal sample sets in each person are represented by separate datapoints. Source data are provided as a Source Data file.

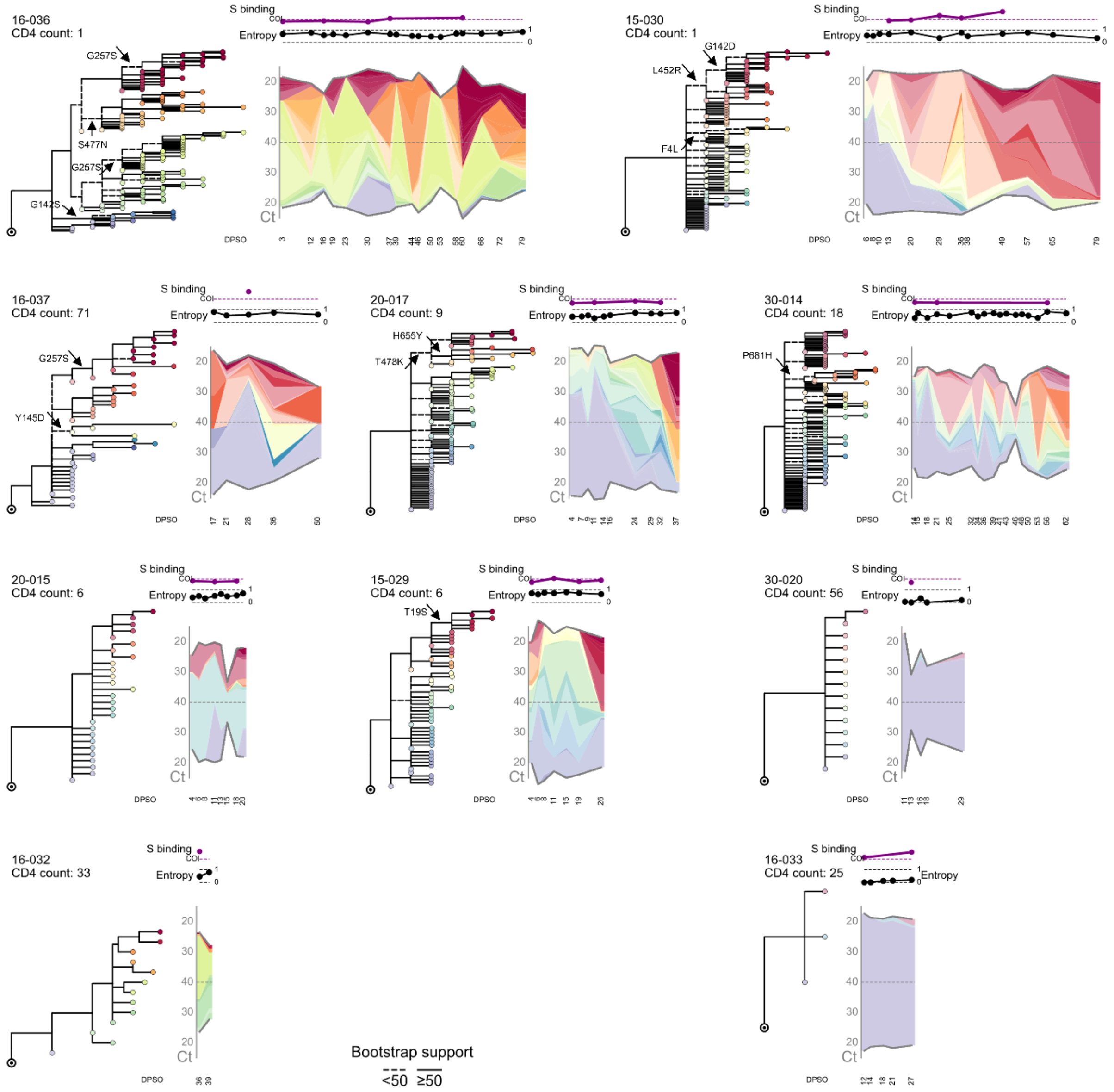

Supplementary Fig. 12. Muller diagrams of haplotype frequencies in PWH with advanced HIV infection. Maximum-likelihood phylogenetic trees rooted on Hu-1 for all haplotypes from each PWH with CD4 counts <200 cells/μL, omitting participants S006-001 and S074-001. Muller diagrams of haplotype frequencies are shown to the right of participant trees. Haplotypes are colored according to the placement of the haplotype on the phylogenetic tree. The estimated population size is shown as proportional to the SARS-CoV-2 RNA levels (rRT-PCR Ct values). Serum antibody binding to spike protein (optical density, 450 nm [OD450]) and normalized Shannon entropy (entropy) are shown above the Muller diagram for each participant. The positivity

cutoff index (COI) of 0.4 for serum antibody binding to spike protein is indicated. Relevant mutations defining clades of haplotypes are indicated on each tree where applicable.

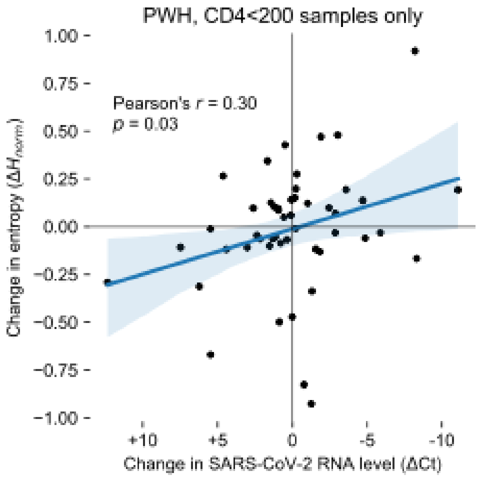

Supplementary Fig. 13. Changes in SARS-CoV-2 RNA levels are correlated with changes in population diversity. Each pair of successive samples within each PWH with CD4 counts <200 cells/μL are shown as a point. Changes in SARS-CoV-2 RNA levels are shown as ΔCt with negative values (i.e., increases in RNA levels) on the right of the origin. Changes in population diversity are shown as the difference in normalized Shannon entropy (H_{norm}). Linear regression results are shown in blue with the shaded region indicating the bootstrapped 95% confidence interval of the mean. Source data are provided as a Source Data file.

Supplementary Table 1. Functional roles of mutations under positive selection in PWH with CD4 counts <200 cells/μL.

Variant	FUBAR p > 0.90	Frequency Change	Infectivity	Immune Evasion	Additional notes
F4L	TRUE	TRUE	Unknown	Unknown	No references
L18F	TRUE	TRUE	No	Yes[3]	
T19I/S	TRUE	TRUE	No[4]	Unknown	Decreased infectivity, unknown impact on neutralization
T22A	TRUE	TRUE	Unknown	Unknown	No references
T95I	TRUE	TRUE	Yes[5]	No	Potentially enables binding to AXL
D138H/N	TRUE	TRUE	Unknown	Unknown	Likely directional selection based on surveillance sequences[6]
RDR2	N/A*	TRUE	No	Yes[7,8]	
G142D/S	TRUE	TRUE	Unknown	Yes[9,10]	
Y145D/H	TRUE	TRUE	Unknown	Yes[11]	
RDR4	N/A*	TRUE	No	Yes[7]	
S247A	TRUE	TRUE	Unknown	Unknown	No references
S255P	TRUE	TRUE	Unknown	Yes[12]	S255F associated with loss of neutralization
G257D/S	TRUE	TRUE	Unknown	Unknown[13,14]	In antigenic supersite; effect on neutralization not significant for some tested NTD mAbs
N370K	TRUE	TRUE	Yes[15]	No	
N440K	TRUE	TRUE	Yes[16]	Yes[17]	
L452R	TRUE	TRUE	Yes[18]	Yes[8]	
S477N	TRUE	TRUE	Yes[19]	Yes[8]	
E484K	TRUE	TRUE	No	Yes[8,20]	
S494P/T	TRUE	TRUE	Yes[8]	Yes[8]	
N501Y/T	TRUE	TRUE	Yes[8,21]	Yes[8]	
H655Y	TRUE	TRUE	Yes[22]	No[23]	Improved cleavage and fusogenicity
Q675R/L	TRUE	TRUE	Yes[24]	No	
P681R/H	TRUE	TRUE	Yes[25,26]	Yes[26]	Escapes IFITM Restriction
A706V	TRUE	TRUE	Unknown	Unknown	No references
T716I	TRUE	TRUE	No[27]	No	Destabilization effect
N764K	TRUE	TRUE	Yes[4]	Yes[4]	
G769V	TRUE	TRUE	Unknown	Unknown	Sporadic appearance, sometimes with E484K[28]
D950N	TRUE	TRUE	Yes[29]	No	
A1078V	TRUE	TRUE	Unknown	Unknown	Also observed in[30]
E1092K	TRUE	TRUE	Unknown[31]	No	Improved stability of trimer
P1069S	TRUE	TRUE	Unknown	No	
G1219V	TRUE	TRUE	Unknown	Unknown	Sporadic appearance globally[32]

Pr(PS): Probability of positive selection
*Indels not applicable to analysis with FUBAR

Note to Supplementary Table 1

Mutations detected as under positive selection (determined as in Methods) in at least one participant are shown. Presumptive roles in infectivity and immune evasion were assessed by literature review as of September 2023. Immune evasion was defined as evidence of escape from any monoclonal antibody or from polyclonal sera; infectivity was defined as increased receptor binding, increased fusogenicity, or other growth advantage in vitro. Recurrently-deleted regions RDR2 and RDR4 were included in the analysis despite being ineligible for dN/dS calculation via

FUBAR because they were repeatedly observed in PWH with CD4 counts <200 cells/μL (see Fig. 3b) and were associated with longitudinal intra-host frequency increases >20%.

Supplementary references

1 Balaban, M., Moshiri, N., Mai, U., Jia, X. & Mirarab, S. TreeCluster: Clustering biological sequences using phylogenetic trees. *PLOS ONE* **14**, e0221068 (2019). https://doi.org:10.1371/journal.pone.0221068

2 Lam, H. M., Ratmann, O. & Boni, M. F. Improved Algorithmic Complexity for the 3SEQ Recombination Detection Algorithm. *Mol Biol Evol* **35**, 247-251 (2018). https://doi.org:10.1093/molbev/msx263

3 McCallum, M. *et al.* N-terminal domain antigenic mapping reveals a site of vulnerability for SARS-CoV-2. *Cell* **184**, 2332-2347 e2316 (2021). https://doi.org:10.1016/j.cell.2021.03.028

4 Pastorio, C. *et al.* Determinants of Spike infectivity, processing, and neutralization in SARS-CoV-2 Omicron subvariants BA.1 and BA.2. *Cell Host Microbe* **30**, 1255-1268 e1255 (2022). https://doi.org:10.1016/j.chom.2022.07.006

5 Wilkinson, S. A. J. *et al.* Recurrent SARS-CoV-2 mutations in immunodeficient patients. *Virus Evol* **8**, veac050 (2022). https://doi.org:10.1093/ve/veac050

6 Nunes, D. R., Braconi, C. T., Ludwig-Begall, L. F., Arns, C. W. & Duraes-Carvalho, R. Deep phylogenetic-based clustering analysis uncovers new and shared mutations in SARS-CoV-2 variants as a result of directional and convergent evolution. *PLoS One* **17**, e0268389 (2022). https://doi.org:10.1371/journal.pone.0268389

7 McCarthy, K. R. *et al.* Recurrent deletions in the SARS-CoV-2 spike glycoprotein drive antibody escape. *Science* (2021). https://doi.org:10.1126/science.abf6950

8 Harvey, W. T. *et al.* SARS-CoV-2 variants, spike mutations and immune escape. *Nat Rev Microbiol* **19**, 409-424 (2021). https://doi.org:10.1038/s41579-021-00573-0

9 Tzou, P. L., Tao, K., Pond, S. L. K. & Shafer, R. W. Coronavirus Resistance Database (CoV-RDB): SARS-CoV-2 susceptibility to monoclonal antibodies, convalescent plasma, and plasma from vaccinated persons. *PLoS One* **17**, e0261045 (2022). https://doi.org:10.1371/journal.pone.0261045

10 Shen, L. *et al.* Spike Protein NTD mutation G142D in SARS-CoV-2 Delta VOC lineages is associated with frequent back mutations, increased viral loads, and immune evasion. *Preprint at https://www.medrxiv.org/content/10.1101/2021.09.12.21263475v1* (2021). https://doi.org:10.1101/2021.09.12.21263475

11 Haslwanter, D. *et al.* A Combination of Receptor-Binding Domain and N-Terminal Domain Neutralizing Antibodies Limits the Generation of SARS-CoV-2 Spike Neutralization-Escape Mutants. *mBio* **12**, e02473-02421 (2021). https://doi.org:10.1128/mbio.02473-21

12 Mathema, B. *et al.* Genomic Epidemiology and Serology Associated with a SARS-CoV-2 R.1 Variant Outbreak in New Jersey. *mBio* **13**, e02141-02122 (2022). https://doi.org:10.1128/mbio.02141-22

13 Wang, Q. *et al.* Antigenic characterization of the SARS-CoV-2 Omicron subvariant BA.2.75. *Cell Host Microbe* **30**, 1512-1517 e1514 (2022). https://doi.org:10.1016/j.chom.2022.09.002

14 Cerutti, G. *et al.* Potent SARS-CoV-2 neutralizing antibodies directed against spike N-terminal domain target a single supersite. *Cell Host Microbe* **29**, 819-833 e817 (2021). https://doi.org:10.1016/j.chom.2021.03.005

15 Zhang, Y. *et al.* Cross-species tropism and antigenic landscapes of circulating SARS-CoV-2 variants. *Cell Rep* **38**, 110558 (2022). https://doi.org:10.1016/j.celrep.2022.110558

16 Tandel, D., Gupta, D., Sah, V. & Harshan, K. H. N440K variant of SARS-CoV-2 has Higher Infectious Fitness. *Preprint at https://www.biorxiv.org/content/10.1101/2021.04.30.441434v1.abstract* (2021). https://doi.org:10.1101/2021.04.30.441434

17 Liu, L. *et al.* Striking antibody evasion manifested by the Omicron variant of SARS-CoV-2. *Nature* **602**, 676-681 (2022). https://doi.org:10.1038/s41586-021-04388-0

18 Zhang, Y. *et al.* SARS-CoV-2 spike L452R mutation increases Omicron variant fusogenicity and infectivity as well as host glycolysis. *Signal Transduct Target Ther* **7**, 76 (2022). https://doi.org:10.1038/s41392-022-00941-z

19 Singh, A., Steinkellner, G., Kochl, K., Gruber, K. & Gruber, C. C. Serine 477 plays a crucial role in the interaction of the SARS-CoV-2 spike protein with the human receptor ACE2. *Sci Rep* **11**, 4320 (2021). https://doi.org:10.1038/s41598-021-83761-5

20 Jangra, S. *et al.* SARS-CoV-2 spike E484K mutation reduces antibody neutralisation. *Lancet Microbe* **2**, e283-e284 (2021). https://doi.org:10.1016/S2666-5247(21)00068-9

21 Cubuk, H. & Ozbi, L. M. In silico analysis of SARS-CoV-2 spike protein N501Y and N501T mutation effects on human ACE2 binding. *J Mol Graph Model* **116**, 108260 (2022). https://doi.org:10.1016/j.jmgm.2022.108260

22 Escalera, A. *et al.* Mutations in SARS-CoV-2 variants of concern link to increased spike cleavage and virus transmission. *Cell Host Microbe* **30**, 373-387 e377 (2022). https://doi.org:10.1016/j.chom.2022.01.006

23 Willett, B. J. *et al.* SARS-CoV-2 Omicron is an immune escape variant with an altered cell entry pathway. *Nat Microbiol* **7**, 1161-1179 (2022). https://doi.org:10.1038/s41564-022-01143-7

24 Arora, P. *et al.* Functional analysis of polymorphisms at the S1/S2 site of SARS-CoV-2 spike protein. *PLoS One* **17**, e0265453 (2022). https://doi.org:10.1371/journal.pone.0265453

25 Liu, Y. *et al.* Delta spike P681R mutation enhances SARS-CoV-2 fitness over Alpha variant. *Cell Rep* **39**, 110829 (2022). https://doi.org:10.1016/j.celrep.2022.110829

26 Lista, M. J. *et al.* The P681H Mutation in the Spike Glycoprotein of the Alpha Variant of SARS-CoV-2 Escapes IFITM Restriction and Is Necessary for Type I Interferon Resistance. *Journal of Virology* **96**, 01250-01222 (2022). https://doi.org:10.1128/jvi.01250-22

27 Magazine, N. *et al.* Mutations and Evolution of the SARS-CoV-2 Spike Protein. *Viruses* **14** (2022). https://doi.org:10.3390/v14030640

28 Hirotsu, Y. & Omata, M. Detection of R.1 lineage severe acute respiratory syndrome coronavirus 2 (SARS-CoV-2) with spike protein W152L/E484K/G769V mutations in Japan. *PLoS Pathog* **17**, e1009619 (2021). https://doi.org:10.1371/journal.ppat.1009619

29 Furusawa, Y. *et al.* In SARS-CoV-2 delta variants, Spike-P681R and D950N promote membrane fusion, Spike-P681R enhances spike cleavage, but neither substitution affects pathogenicity in hamsters. *EBioMedicine* **91**, 104561 (2023). https://doi.org:10.1016/j.ebiom.2023.104561

30 Harari, S. *et al.* Drivers of adaptive evolution during chronic SARS-CoV-2 infections. *Nat Med* **28**, 1501-1508 (2022). https://doi.org:10.1038/s41591-022-01882-4

31 Bascos, N. A. D., Mirano-Bascos, D. & Saloma, C. P. Structural Analysis of Spike Protein Mutations in the SARS-CoV-2 P.3 Variant. *Preprint at https://www.biorxiv.org/content/10.1101/2021.03.06.434059v1* (2021). https://doi.org:10.1101/2021.03.06.434059

32 Colson, P. *et al.* Spreading of a new SARS-CoV-2 N501Y spike variant in a new lineage. *Clin Microbiol Infect* **27**, 1352 e1351-1352 e1355 (2021). https://doi.org:10.1016/j.cmi.2021.05.006
