# Supplementary material for: Rapid intra-host diversification and evolution of SARS-CoV-2 in advanced HIV infection
Source: Nat Commun. 2024 Aug 22;15:7240. doi: 10.1038/s41467-024-51539-8 (PMC11341811; doi:10.1038/s41467-024-51539-8)
Supplement: Supplementary file 3 — Description of Additional Supplementary Files [file 41467_2024_51539_MOESM3_ESM.pdf]

**Description of Additional Supplementary Files:**

**Supplementary Data 1.** Characteristics of study participants.

**Supplementary Data 2.** Number of single-genome sequences (SGS) recovered in each sequenced sample.

**Supplementary Data 3.** Whitelisted UMI primer sequences used for HT-SGS validation experiments.
