# Supplementary material for: Rapid intra-host diversification and evolution of SARS-CoV-2 in advanced HIV infection
Source: Nat Commun. 2024 Aug 22;15:7240. doi: 10.1038/s41467-024-51539-8 (PMC11341811; doi:10.1038/s41467-024-51539-8)
Supplement: Supplementary file 7 — Reporting Summary [file 41467_2024_51539_MOESM7_ESM.pdf]

nature portfolio

Corresponding author(s): Eli A. Boritz

Last updated by author(s): Jul 22, 2024

Reporting Summary

Nature Portfolio wishes to improve the reproducibility of the work that we publish. This form provides structure for consistency and transparency in reporting. For further information on Nature Portfolio policies, see our Editorial Policies and the Editorial Policy Checklist.

Statistics

For all statistical analyses, confirm that the following items are present in the figure legend, table legend, main text, or Methods section.

n/a	Confirmed	
☐	☒	The exact sample size (*n*) for each experimental group/condition, given as a discrete number and unit of measurement
☐	☒	A statement on whether measurements were taken from distinct samples or whether the same sample was measured repeatedly
☐	☒	The statistical test(s) used AND whether they are one- or two-sided<br>*Only common tests should be described solely by name; describe more complex techniques in the Methods section.*
☒	☐	A description of all covariates tested
☐	☒	A description of any assumptions or corrections, such as tests of normality and adjustment for multiple comparisons
☐	☒	A full description of the statistical parameters including central tendency (e.g. means) or other basic estimates (e.g. regression coefficient) AND variation (e.g. standard deviation) or associated estimates of uncertainty (e.g. confidence intervals)
☐	☒	For null hypothesis testing, the test statistic (e.g. *F*, *t*, *r*) with confidence intervals, effect sizes, degrees of freedom and *P* value noted<br>*Give P values as exact values whenever suitable.*
☒	☐	For Bayesian analysis, information on the choice of priors and Markov chain Monte Carlo settings
☒	☐	For hierarchical and complex designs, identification of the appropriate level for tests and full reporting of outcomes
☒	☐	Estimates of effect sizes (e.g. Cohen's *d*, Pearson's *r*), indicating how they were calculated

Our web collection on statistics for biologists contains articles on many of the points above.

Software and code

Policy information about availability of computer code

Data collection	UMI-tagged amplicon sequences were collected on a Pacific Biosciences Sequel II sequencer using a 20-hour movie time under circular consensus sequencing (CCS) mode with SMRT Link (v. 11.0.0.146107).
Data analysis	CCS were demultiplexed with lima (v. 2.5.1). Reads were oriented with vsearch (v. 2.21.1). Cutadapt (v. 4.1) was used to trim primer sequences and perform length filtering. Python scripts available in UMI-pacbio-pipeline (v. 1.1; https://github.com/niaid/UMI-pacbio-pipeline/releases/tag/SC2-HIV-demo) were used to parse and bin reads based on their inferred UMI sequence. UMI bins were clustered with vsearch and consensus single-genome sequences were generated with minimap2 (v. 2.24) and bcftools (v. 1.13). Non-SARS-CoV-2 sequences were removed with blastn (v. 2.9.0). Python scripts in UMI-pacbio-pipeline were used to filter final sequences and call haplotypes. Haplotype entropy was computed with a Perl script, and Jensen-Shannon distance was computed via the 'jensenshannon' method in SciPy (v. 1.8.1). Recombinant haplotypes were identified with 3SEQ (v. 1.8.0). To perform phylogenetic inference, indels were encoded via 2matrix (v. 1.0) and ML tree reconstruction with bootstrapping was performed with iqtree (v. 1.6.12). Phylogenetic clades were identified with TreeCluster (v. 1.0.3). Hypothesis testing of selection was performed with FUBAR in HyPhy (v. 2.5.46). GraphPad Prism (v. 9.4.0) was used for statistical testing and plotting of datapoints. Phylogenetic trees were plotted with EvolView (v. 3.0) and Biopython (v. 1.79).

For manuscripts utilizing custom algorithms or software that are central to the research but not yet described in published literature, software must be made available to editors and reviewers. We strongly encourage code deposition in a community repository (e.g. GitHub). See the Nature Portfolio guidelines for submitting code & software for further information.

Data

Policy information about availability of data

All manuscripts must include a data availability statement. This statement should provide the following information, where applicable:

- Accession codes, unique identifiers, or web links for publicly available datasets
- A description of any restrictions on data availability
- For clinical datasets or third party data, please ensure that the statement adheres to our policy

All data that support the findings of this study are available in this article and supplementary materials. Long-read sequencing data that support the findings of this study have been deposited in the NCBI SRA database with accession codes SRR27325889-SRR27326072 under BioProject PRJNA1055920 [https://www.ncbi.nlm.nih.gov/bioproject/?term=PRJNA1055920]. Source data are provided with this paper.

Research involving human participants, their data, or biological material

Policy information about studies with human participants or human data. See also policy information about sex, gender (identity/presentation), and sexual orientation and race, ethnicity and racism.

Reporting on sex and gender	Information on biological sex was collected in these studies through self-reporting. While sex was considered as a potential exposure of interest in the design of the original studies, analysis was not stratified by sex in the current study. Out of 47 individuals enrolled in this study, 28 were female,18 were male, and one was unknown (not reported). These details are described in Supplementary Data 1.
Reporting on race, ethnicity, or other socially relevant groupings	No subgroup analysed on sex, gender, race, ethnicity or other socially relevant groupings.
Population characteristics	Population characteristics for respiratory specimens sequenced via HT-SGS were described in the Supplementary Data 1.
Recruitment	Recruitment of study participants was performed in compliance with relevant ethical regulations. Participants provided informed consent before study.<br>The hospitalized inpatient cohort was enrolled from 20 sentinel surveillance hospitals in 8 of the 9 South African provinces. Enrolments were limited to individuals living within a 50-kilomenter radius of the respective hospitals. Samples were collected at enrolment and every second day thereafter until cessation of shedding. All specimens were transported to the National Institute for Communicable Disease (NICD) in Johannesburg for processing and testing (Meiring et al 2022, Clin Infect Dis).<br>The outpatient cohort was enrolled at two sites, Klerksdorp (North West Province) and Soweto (Gauteng Province), South Africa. To collect samples, study staff visited 3 times a week up for six weeks. Of two swabs collected, one of which was transported to the National Institute for Communicable Diseases (NICD) for SARS-CoV-2 RNA detection (Kleynhans et al 2023, Clin Infect Dis).<br>As participation in these studies required participants to have sought and/or accepted engagement with local health care systems, it is possible that participation was associated with some degree of health-care-seeking. Future studies will be required to understand any potential differences in findings that might be made among individuals who do not engage with local health care systems.
Ethics oversight	All participants provided written informed consent to be enrolled in the respective studies. For the hospitalized cohort, ethical clearance was obtained through the University of the Witwatersrand health research ethics committees (HREC) (Medical) (M160667); Stellenbosch University HREC (15206); University of Pretoria HREC (256/2020), and University of the Free State HREC (HSD2020/0625). For the outpatient cohort, clearance was obtained from the University of the Witwatersrand HREC (M2008114) (e.g. Meiring et al 2022, Clin Infect Dis; Kleynhans et al 2023, Clin Infect Dis).

Note that full information on the approval of the study protocol must also be provided in the manuscript.

Field-specific reporting

Please select the one below that is the best fit for your research. If you are not sure, read the appropriate sections before making your selection.

☒ Life sciences ☐ Behavioural & social sciences ☐ Ecological, evolutionary & environmental sciences

For a reference copy of the document with all sections, see nature.com/documents/nr-reporting-summary-flat.pdf

Life sciences study design

All studies must disclose on these points even when the disclosure is negative.

Sample size	No formal sample size calculation was performed. Respiratory specimens from individuals who had participated in previous cohort studies of people with COVID-19 were selected for this exploratory study (e.g. Meiring et al 2022, Clin Infect Dis; Kleynhans et al 2023, Clin Infect Dis). SARS-CoV-2 spike gene was characterized in 184 samples from 22 people with HIV and 25 people without HIV.
Data exclusions	J039-007 exhibited abnormally high value in one of the three spike diversity measurements, and S074-001 appeared to be infected by two different variants, wherein recombinant haplotypes were detected. As these features could distort the result of spike diversity, the data from

two individuals (J039-007 and S074-001) were excluded from the diversity assessment.

Replication	HT-SGS for SARS-CoV-2 spike gene was performed twice for 128 samples which had high viral load, whereas the remaining samples were sequenced once due to insufficient amounts of viral RNA. In the test of HT-SGS performance with SARS-CoV-2 spike gene from various studies, repeating the runs produced reproducible sequencing data.
Randomization	No randomization was performed. We analysed all available data.
Blinding	No blinding was performed. We analysed all available data.

Reporting for specific materials, systems and methods

We require information from authors about some types of materials, experimental systems and methods used in many studies. Here, indicate whether each material, system or method listed is relevant to your study. If you are not sure if a list item applies to your research, read the appropriate section before selecting a response.

Materials & experimental systems

n/a	Involved in the study	
☐	☒	Antibodies
☒	☐	Eukaryotic cell lines
☒	☐	Palaeontology and archaeology
☒	☐	Animals and other organisms
☒	☐	Clinical data
☒	☐	Dual use research of concern
☒	☐	Plants

Methods

n/a	Involved in the study	
☒	☐	ChIP-seq
☒	☐	Flow cytometry
☒	☐	MRI-based neuroimaging

Antibodies

Antibodies used	CR3022 - produced by South African Medical Research Council Antibody Immunity Research Unit; working dilution 10 ug/mL<br>Palivizumab - produced by Medimmune, RRID: AB_2459638; working dilution 10 ug/mL<br>Anti-human-horseradish-peroxidase - produced by Merck, catalog number A0170-1
Validation	Antibodies were cloned, expressed and quality controlled according to published IC50 or EC50 data.

Plants

Seed stocks	*Report on the source of all seed stocks or other plant material used. If applicable, state the seed stock centre and catalogue number. If plant specimens were collected from the field, describe the collection location, date and sampling procedures.*
Novel plant genotypes	*Describe the methods by which all novel plant genotypes were produced. This includes those generated by transgenic approaches, gene editing, chemical/radiation-based mutagenesis and hybridization. For transgenic lines, describe the transformation method, the number of independent lines analyzed and the generation upon which experiments were performed. For gene-edited lines, describe the editor used, the endogenous sequence targeted for editing, the targeting guide RNA sequence (if applicable) and how the editor was applied.*
Authentication	*Describe any authentication procedures for each seed stock used or novel genotype generated. Describe any experiments used to assess the effect of a mutation and, where applicable, how potential secondary effects (e.g. second site T-DNA insertions, mosiacism, off-target gene editing) were examined.*
